# Supplementary figures and images for: Implementation of a complex intervention to improve care for patients whose situations are clinically uncertain in hospital settings: A multi-method study using normalisation process theory
Source: PLoS One. 2020 Sep 16;15(9):e0239181. doi: 10.1371/journal.pone.0239181 (PMC7494119; doi:10.1371/journal.pone.0239181)

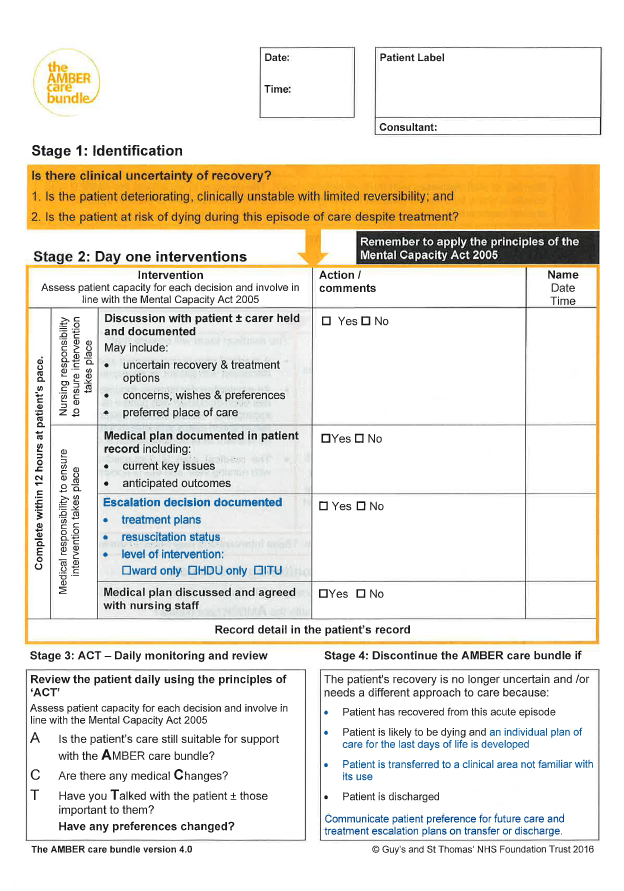

Supplement: S1 Appendix — (DOCX) [file pone.0239181.s001.docx]
