# Supplementary material for: Implementation of a complex intervention to improve care for patients whose situations are clinically uncertain in hospital settings: A multi-method study using normalisation process theory
Source: PLoS One. 2020 Sep 16;15(9):e0239181. doi: 10.1371/journal.pone.0239181 (PMC7494119; doi:10.1371/journal.pone.0239181)
Supplement: S2 Appendix — (DOCX) [file pone.0239181.s002.docx]

# DRAFT TOPIC GUIDE FOR QUALITATIVE INTERVIEW WITH PATIENT PARTICIPANT

Introductory questions

I’d like to start this interview by asking if you would tell me the story, in any way you choose, about what happened to bring you to hospital.

A - ILLNESS RELATED QUESTIONS

I’d like you to tell me about your illness and how it has been bothering or troubling you whilst you have been in hospital. Probe by asking participant about symptoms and associated distress he or she has been experiencing

Tell me in your own words how you have been feeling in yourself

Tell me how the doctors and nurses caring for you have helped manage all these issues.

B - INFORMATION AND COMMUNICATION ISSUES

I’d like to ask you about other important aspects of care while you have been in hospital, for example what you have been told about your illness and treatment?

Did you completely understand what the doctors and nurses told you? What wasn’t clear to you?

Did you feel that you were getting clear and consistent information from the nurses and doctors?

Were you encouraged by the doctors and nurses to ask questions about your situation? Ask for examples.

In your opinion to what extent do you feel the doctors and nurses have listened to your concerns? What more could they have done, and why?

C - INVOLVEMENT IN DECISION MAKING

I’d like to explore with you to what extent you and your family has felt involved in making important decisions about your care and treatment.

Can you give me example about issues where this has taken place?

How did you and your family feel about this? What more, if anything, would you have liked to talk about, and why?

Have the doctors and nurses caring for you talked about the future? What did they discuss? How did you and your family feel about this?

What more, if anything, would you have liked to talk about, and why?

D - CONFIDENCE IN CARE PROVIDED

In what ways do you feel confident about the care and treatment the doctors and nurse have given you? Probe for examples and areas where participant does not feel confident, and why.

Do you feel that the doctors, nurses and other health professionals (e.g. Physio, OT) worked well together? Give examples if possible.

E - CONCLUSION OF INTERVIEW

Is there anything else you would like to tell me about your illness or your time in hospital?

# DRAFT TOPIC GUIDE FOR QUALITATIVE INTERVIEW WITH RELATIVE/FRIEND PARTICIPANT

Introductory questions

I’d like to start this interview by asking if you would tell me the story, in any way you choose, of what happened leading to your relative/friend’s admission to hospital.

A- Illness related questions

I’d like you to tell me what you understand about your relative/friend’s illness and how it has been bothering or troubling them while he/she has been in hospital. *Probe by asking participant about symptoms and associated distress their relative/friend has been experiencing*

Tell me the main problem/s your relative/friend has been experiencing.

Tell me if you can how the doctors and nurses caring for your relative/friend have helped manage these problem/s.

B- Information and communication issues

I’d like to ask you about other important aspects of care while your relative/friend has been in hospital, for example what you have been told about your relative/friend’s illness and treatment?

Did you completely understand what the doctors and nurses told you? If you did not tell me what was unclear?

Did you feel that you were getting consistent information from the nurses and doctors? Give examples. If not, in what ways was information different or unclear from one health care profession to the next, or at different times?

Were you encouraged by the doctors and nurses to ask questions about your relative/friend’s situation? Ask for examples. If you were not would you have liked to? Why?

In your opinion to what extent do you feel they have listened to your concerns? Do you think thy ewer taken seriously and then followed up? Give examples.

C- Involvement in decision making

I’d like to explore with you to what extent you have felt involved in making important decisions together with your relative/friend’s relating to care and treatment?

Can you give me example/s about issues where this took place?

How did you and your relative/friend feel about this? What more, if anything, would you have liked to talk about, and why?

Did the doctors and nurses caring for your relative/friend talk about the future? What did they discuss? How did you respond to this?

What more, if anything, would you have liked to talk about, and why?

D- Confidence in care provided

In what ways do you feel confident about the care and treatment the doctors and nurse have given your relative/friend? *Probe for examples and areas where participant did not feel confident, and why*

Do you feel that the doctors, nurses and other health professionals (e.g. Physio, OT) worked well together? Give example/s if possible.

E- Conclusion of interview

Is there anything else you would like to tell me about your relative/friend’s illness or their time in hospital?
