# Supplementary material for: Implementation of a complex intervention to improve care for patients whose situations are clinically uncertain in hospital settings: A multi-method study using normalisation process theory
Source: PLoS One. 2020 Sep 16;15(9):e0239181. doi: 10.1371/journal.pone.0239181 (PMC7494119; doi:10.1371/journal.pone.0239181)
Supplement: S3 Appendix — (DOC) [file pone.0239181.s003.doc]

# DRAFT TOPIC GUIDE FOR FOCUS GROUPS WITH HEALTHCARE PROFESSIONAL PARTICIPANTS

A. UNDERSTANDING AND VALUE OF THE INTERVENTION

- I would be grateful if you would tell me what you feel you and your colleagues feel about the AMBER care bundle.
- What do you consider to be the benefits from the AMBER care bundle?
- What do you consider to be the harms from the AMBER care bundle?
- In what ways do you think these conversations and plans that are then made produce the outcomes that are important to patient and their families?
- What aspects do you find difficult about caring for these patients? Can you give examples?
  - Probe: How do you recognize patients who are deteriorating and there is uncertainty as to their recovery or continued decline leading eventually to end of life.
  - Probe: How do you differentiate between this group and people who you consider are actively dying?

B. ENGAGEMENT WITH THE INTERVENTION

- How well do you feel health care professionals work together in this sort of situation?
  - What is done well? What could be done better? Give examples where possible.
- Can you tell about what leadership there is or champions who can support you when you are managing the care of a patient whose clinical situation is uncertain?
- How does staff turnover on the ward influence how the team works together?

C. DELIVERY OF THE INTERVENTION

- Can you explain to me how you and your colleagues talk to patients and their families about their situation? – What does this lead to?
  - Probe: How do you find this?
  - Probe: How are you supported with this? Prompt: Is there a system in place on the ward for providing emotional support to members of the healthcare team?
- How frequently and how long are conversations that take place with patients and their families?

D. ACCEPTIBALITY, AND POTENTIAL MODIFICATIONS

- To what extent do you think the AMBER care bundle needs to be refined or adapted to make it more acceptable/more relevant to the patients you are caring for and their families?
- I would like you to consider the different aspects of the AMBER care bundle - which bits do you think can remain the same and which need to change for the ward you are working on and why?
- Are you in any way unhappy with any aspect of the content or delivery of the AMBER care bundle? What specifically, and why?
- What are your views on the way in which the AMBER care bundle was implemented on this ward? What worked and why and what could have bene done differently, and why?
- I would like you to consider to what extent is the right amount of the AMBER care bundle getting to the right recipients in the right way?
- Do you think those who are delivering/supporting patient with the AMBER care bundle on this ward adhere to how it was explained and according to the manual? If not, in what ways?
