# Supplementary material for: Implementation of a complex intervention to improve care for patients whose situations are clinically uncertain in hospital settings: A multi-method study using normalisation process theory
Source: PLoS One. 2020 Sep 16;15(9):e0239181. doi: 10.1371/journal.pone.0239181 (PMC7494119; doi:10.1371/journal.pone.0239181)
Supplement: S1 Table — (DOCX) [file pone.0239181.s004.docx]

**S1 Table. Demographics of health professionals involved in focus groups at each site**

| Site | Site 1  *(N=11)* | Site 2  *(N=15)* |
| --- | --- | --- |
| Specialties in involved | Geriatrics | Respiratory |
| Professionals involved (Gender) | Consultant Geriatrician-Ward X (F)  Consultant Geriatrician-Ward Y (M)  Ward Clerk-Ward Y (F)  Ward sister-Ward Y (F)  Ward manager (F)  Ward manager assistant (F)  Physician Associate-Ward X (F)  Matron-Ward X (M)  Nurse assistant (M)  Research nurse (F)  Research nurse (F) | Junior Ward Sister (F)  Staff nurse (F)  Registrar (F)  Senior house office (F)  F1 (F)  Senior house office (F)  Junior doctor (M)  Matron (F)  Palliative Care CNS (F)  Research nurse (F)  Ward manager (F)  Junior doctor (M)  Senior house office (F)  Registrar (M)  F1 (M) |
| Duration | 50 minutes | 49 minutes |
