# Supplementary material for: Implementation of a complex intervention to improve care for patients whose situations are clinically uncertain in hospital settings: A multi-method study using normalisation process theory
Source: PLoS One. 2020 Sep 16;15(9):e0239181. doi: 10.1371/journal.pone.0239181 (PMC7494119; doi:10.1371/journal.pone.0239181)
Supplement: S2 Table — (DOCX) [file pone.0239181.s005.docx]

**SS2 Table. Demographics of patients and carers involved in qualitative interviews at each site**

|  | Site 1 | Site 2 |
| --- | --- | --- |
| Interviews | 8 | 4 |
| Interview participants  Patient  Carer or relative | 0  8 | 2  2 |
| Interview participant ethnicity  White British | 8 | 4 |
| Interview participant gender  Female  Male | 1  2 | 7  2 |
| Relationship with the patient  Wife  Husband  Daughter  Son | 1  1  7  1 | 1  1  0  0 |
| Patient disease group  Cancer  Non-cancer | 1  7 | 0  4 |
| Patient age (years)  50-64  65-79  80-94  95-109  Mean  Median  Range | 0  0  5  3  92  92  85-100 | 1  3  0  0  69  69  63-74 |
| Income  Living comfortably with present income  Coping on present income  Difficult on present income  Prefer not to say | 4  1  1  2 | 1  2  1  0 |
| Pension  State pension  Attendance allowance  Registered disabled | 6  2  0 | 3  0  1 |
