# Supplementary material for: Implementation of a complex intervention to improve care for patients whose situations are clinically uncertain in hospital settings: A multi-method study using normalisation process theory
Source: PLoS One. 2020 Sep 16;15(9):e0239181. doi: 10.1371/journal.pone.0239181 (PMC7494119; doi:10.1371/journal.pone.0239181)
Supplement: S3 Table — (DOCX) [file pone.0239181.s006.docx]

**S3 Table. Demographics of health professionals involved in non-participant observations at each site**

| Site | Site 1 | Site 2 |
| --- | --- | --- |
| Number of meetings observed | 6 *(3 per ward)* | 3 |
| Specialties in involved | Geriatrics | Respiratory |
| Type of meeting | Morning handover | Board round/Morning handover |
| Professionals involved | Consultants  Registrars  SHOs  F2s  F1s  GP trainee  Ward managers  Ward sisters  Ward matron  Occupational therapists  Physiotherapists  Physician associates and trainees | Consultants  Registrars  F1s  Ward sisters  Occupational therapists  Physiotherapists  Discharge coordinator  Palliative Care CNS  Research Nurse  Staff nurse  Respiratory nurse  Nurse facilitator |
| Number of participants per meeting (average) | 7 | 10 |
| Duration | 40 minutes | 30-40 minutes |
