# Supplementary material for: Implementation of a complex intervention to improve care for patients whose situations are clinically uncertain in hospital settings: A multi-method study using normalisation process theory
Source: PLoS One. 2020 Sep 16;15(9):e0239181. doi: 10.1371/journal.pone.0239181 (PMC7494119; doi:10.1371/journal.pone.0239181)
Supplement: S4 Table — (DOCX) [file pone.0239181.s007.docx]

**S4 Table. Demographics of patient participants involved in clinical case note review n, (%)**

|  | | Site 1  (N=20) | Site 2  (N=9) |
| --- | --- | --- | --- |
| Gender | Male | 8 (40.0) | 3 (33.3) |
|  | Female | 12 (60.0) | 6 (66.7) |
| Age | 50-64 | 0 | 1 (11.1) |
|  | 65-79 | 2 (10.0) | 5 (55.6) |
|  | 80+ | 18 (90.0) | 3 (33.3) |
|  | Mean (SD) | 89.0 (5.7) | 77.1 (12.0) |
| Education | Did not go to school | 0 | 0 |
|  | Secondary school (GCSE/O Level) | 9 (45.0) | 3 (33.3) |
|  | Secondary school (A Level) | 5 (25.0) | 4 (44.4) |
|  | Vocational qualification | 1 (5.0) | 1 (11.1) |
|  | University | 4 (20.0) | 0 |
|  | Prefer not to say | 0 | 1 (11.1 |
|  | *Missing* | *1 (5.0)* | *0* |
| Marital status | Single | 4 (20.0) | 0 |
|  | Widowed | 14 (70.0) | 3 (33.3) |
|  | Married/civil partnership/long-term relationship | 2 (10.0) | 6 (66.7) |
| Ethnicity | White British | 19 (95.0) | 9 (100.0) |
|  | Other white | 1 (5.0) | 0 |
| Income | Living comfortably at present | 8 (40.0) | 4 (44.4) |
|  | Coping on present income | 5 (25.0) | 4 (44.4) |
|  | Difficult on present income | 4 (20.0) | 1 (11.1) |
|  | Very difficult on present income | 0 | 0 |
|  | Prefer not to say | 2 (10.0) | 0 |
|  | Don’t know | 1 (5.0) | 0 |
